# Plant sex affects plant-microbiome assemblies of dioecious *Populus cathayana* trees under different soil nitrogen conditions

**DOI:** 10.1186/s40168-022-01387-9

**Published:** 2022-11-05

**Authors:** Qingxue Guo, Lin Liu, Jiantong Liu, Helena Korpelainen, Chunyang Li

**Affiliations:** 1 College of Life and Environmental Sciences, Hangzhou Normal University, Hangzhou, 311121 China; 2 Department of Agricultural Sciences, Viikki Plant Science Centre, University of Helsinki, P.O. Box 27, FI-00014 Helsinki, Finland; 3 College of Agriculture and Biotechnology, Zhejiang University, Hangzhou, 310058 China

**Keywords:** Dioecious plants, Plant-soil continuum, Plant microbiome, Endophytes, Plant-microbe interactions

## Abstract

**Background:**

Dioecious plants have coevolved with diverse plant microbiomes, which are crucial for the fitness and productivity of their host. Sexual dimorphism in morphology, physiology, or gene expression may relate to different microbial compositions that affect male and female fitness in different environments. However, sex-specific impacts on ecological processes that control the microbiome assembly are not well known. In this study, *Populus cathayana* males and females were planted in different nitrogen conditions. It was hypothesized that males and females differently affect bacterial and fungal communities in the rhizosphere soil, roots, old leaves, and young leaves. Physiological traits and transcriptome profiles of male and female plants were investigated to reveal potential mechanisms that control the microbiome assembly.

**Results:**

Our results showed strong niche differentiation that shapes microbial communities leading to a rapid loss of diversity along a decreasing pH gradient from the rhizosphere soil to leaves. Sex had different impacts on the microbial assembly in each niche. Especially fungal endophytes showed great differences in the community structure, keystone species, and community complexity between *P. cathayana* males and females. For example, the fungal co-occurrence network was more complex and the alpha diversity was significantly higher in young female leaves compared to young male leaves. Transcriptome profiles revealed substantial differences in plant-pathogen interactions and physiological traits that clearly demonstrated divergent internal environments for endophytes inhabiting males and females. Starch and pH of young leaves significantly affected the abundance of Proteobacteria, while tannin and pH of roots showed significant effects on the abundance of Chloroflexi, Actinobacteria, and Proteobacteria, and on the bacterial Shannon diversity.

**Conclusion:**

Our results provided important knowledge for understanding sexual dimorphism that affects microbial assemblies, thus advancing our understanding of plant-microbiome interactions.

Video Abstract

**Supplementary Information:**

The online version contains supplementary material available at 10.1186/s40168-022-01387-9.

## Introduction

Dioecious angiosperms, including 15,600 species in 987 genera and 175 families, have evolved independently from hermaphroditic ancestors [1]. Genetic sex determination mechanisms that lead to separate male and female plants have been identified [2]. Males and females have sex-specific adaptions to different environments resulting in biased sex ratios in populations [3–5]. Sexual dimorphism in morphology, physiology, and gene expression, and biased sex ratios are due to selective pressures acting differently on males and females [6–8]. *Populus* species are widely distributed, and they are suitable woody model species to study sex-specific responses of dioecious plants to different environments [5]. Previous studies have provided convincing evidence of male poplars having a better capacity to forage limited nutrients in infertile soil and a higher resistance to other abiotic stresses when compared to female poplars [5, 7, 8].

Plant microbiota or microbiome comprises diverse and complex microbial communities containing bacteria, archaea, and fungi living on the plant surface (e.g., rhizosphere and phyllosphere) as well as inside plants (endosphere) [9–11]. Plants and their microbial inhabitants have coevolved over millions of years and are linked to plant fitness as a “holobiont” [12]. Recent studies suggest that host health, growth, nutrient acquisition, and plant microbiota assembly are largely impacted by complex interactions between plants, microbes, and environments [11–13]. However, the underlying ecological processes in regulating plant-microbiome-environment interactions are still poorly understood.

Studies on dioecious plants have reported sex-specific responses to arbuscular mycorrhizal fungi suggesting that female growth and reproduction benefit more from arbuscular mycorrhizal fungi when compared to males [14]. However, a higher colonization rate or hyphal biomass of arbuscular mycorrhizal fungi in male plants have been suggested to be crucial for males for the maintenance of a higher resistance or tolerance to different abiotic stresses [7, 15]. For example, a closer relationship between arbuscular mycorrhizal fungi and the root system of *P. cathayana* males has been suggested to enhance the capacity of phosphorus forage compared to females [7]. A selective enrichment of a specific microbiome that contributes to stress resistance is driven by variation in plant metabolism and immune-related characters [16, 17]. However, sex-specific plant microbiomes have not been investigated, whereas differences in defense, growth, and reproduction between male and female plants have been well studied [6, 18–20].

The microbial community assembly of the rhizosphere is largely determined by different plant species or genotypes through sensing and responding to root-derived signals [21]. A recent study revealed that *Populus euphratica* males and females harbor sex-specific bacterial and fungal communities in the rhizosphere soil of natural forests [22]. It has been reported that a rapid loss of diversity from soil to roots and then to leaves and flowers indicate a strong selective pressure along the soil-plant continuum [10, 11, 23]. The microorganisms overcome or escape the host’s immune system and successfully colonize plant compartments (endosphere), such as roots, stems, or leaves without causing any disease. The community assembly of endophytes largely depends on the plant species, genotype, and development stage because of differences in plants’ physiology and metabolism [11, 19, 24]. Sexual differences in morphology, physiology, and metabolism between males and females are supposed to lead to distinct plant endophytes. The metabolism of different plant compartment niches generates chemically distinct environments and each host tissue provides a stable availability of metabolites for associated endophytes to maintain the biosynthesis of diverse compounds [23, 25]. Changes in plant metabolism caused by varied environmental conditions are crucial factors that affect microbial communities within plants.

As an important dioecious plant, *P. cathayana* has been extensively studied for its responses to different environmental conditions. It has been discovered that the sexual differences are greatest in poor environments [5]. *P. cathayana* males have higher carbon fixation and transportation from a carbon source (leaf) to sinks, whereas *P. cathayana* females show higher secondary metabolic activities and glycolysis, but a lower expression of resistance genes under soil nitrogen-limited conditions [8, 20, 26]. In internal plant compartments, such as root and leaf tissues, a fine-tuning and adaptation of the microbiome is clearly evident [9], but the specific factors controlling the local microbial assembly and stability are still unclear. In the present study, the effects of internal traits on the microbial assembly and functions in *P. cathayana* males and females were explored. Three soil nitrogen levels were used to cultivate plant materials, and sampled rhizosphere soils, roots, old leaves, and young leaves. We firstly hypothesized that niche-dependent bacterial and fungal communities occur along the soil-plant continuum. Then, sex would impose distinct impacts on the bacterial and fungal communities in each niche. As previously shown, *P. tremula* females prioritize nutrient acquisition, and flavonoid and condensed tannin production, while males allocate more to the growth of aerial parts under limited nutrient conditions [18]. Therefore, the sex effect on the microbial assembly of endophytes was supposed to be stronger when soil nitrogen level is limited. If the nitrogen level was recovered to a normal level, the sex effect would be expected to decrease.

## Materials and methods

### Experimental setup

*Populus cathayana* forests widely distribute in Qinghai province, China. The original cuttings of *P. cathayana* males and females were sampled from five populations as detailed in a previous study [7]. Five populations were separated by at least 50 km for each other. All cuttings were then cultivated in a greenhouse at the Hangzhou Normal University, Zhejiang Province, China. The whole experiment was conducted in this greenhouse with the temperature ranging between 21 and 25 °C during the day and 15 and 18 °C at night, with 12–14 h photoperiod [7]. Soil with a soil organic matter content of 4 g kg^−1^, total nitrogen content 1.5 g kg^−1^, and pH 7.1 was collected in the garden and homogenized. Then, the collected soil, sand and vermiculite in a 1:1:1 ratio were homogenized to set “nutrient-limited” soil. We sampled 30 male and 30 female cuttings, and planted them in plastic pots (30 cm diameter and 21 cm height), which were filled with nutrient-limited soil at the beginning of March, 2019. Each pot contained one cutting. All plants were irrigated once a week with 150 ml Long Ashton solution containing 2 mM NH_4_NO_3_ until the beginning of April when the cuttings started root formation [27] (see Supplementary methods S1).

In all, 24 male and 21 female cuttings successfully sprouted, of which 15 male and 15 female individuals with a similar height for further experiments were evenly chosen from the five original populations. Three nitrogen treatments through controlling the N content of the Long Ashton solution were designed. The control-nitrogen treatment, re-irrigated with control-nitrogen solution treatment, and limited-nitrogen treatment were named as N1, N2, and N3, respectively. Thus, the experimental setup with two sexes (males and females) and three N treatments (N1, N2, and N3) included six treatments in total. Each treatment contained five replicates. The control-nitrogen treatment was irrigated with a solution containing 2 mM NH_4_NO_3_ (considered as a normal N supply) twice a week till the end of the experiment. Similarly, the limited-nitrogen treatment was controlled by excluding the N source from the solution when irrigated twice a week till end of the experiment. Two steps were used to construct the re-irrigated with control-nitrogen solution treatment. Firstly, 5 male and 5 female individuals were irrigated with 200 ml solution without N source twice a week until mid-July and then re-irrigated with the solution containing 2 mM NH_4_NO_3_ twice a week until end of the experiment.

### Sample collection and soil traits

Rhizosphere soil, roots, and old and young leaves were collected at the beginning of September, 2019. Rhizosphere soil was defined as the soil tightly attached to plant roots. Roots of each plant were sampled. After the N treatment, 2 or 3 new emerging leaves of each plant were labelled and named them as old leaves since they began to be senescent by the end of the experiment. Recent fully expanded leaves of each plant present before sampling were named as young leaves. Both old and young leaves were collected. Soil, roots, old leaves, and young leaves were referred as “microhabitats” in this study. All samples were first kept on ice and stored at −80 °C before extracting DNA in the laboratory. The soil chemical traits including pH, total nitrogen (TN), total phosphorus (TP), available phosphorus (AP), NH_4_^+^, and NO_3_^−^, were measured according to standard protocols [13]. Activities of β-1,4-glucosidase (BG) related to labile-C-cycling and β-1,4-N-acetylglucosaminidase (NAG) related with N-cycling were tested by Elisa kits (Shanghai, China). Soil microbial carbon and nitrogen biomass were determined by the chloroform fumigation extraction method [28].

### DNA extraction and amplification

For the rhizosphere soil, DNA was extracted from 0.5 g soil using the E.Z.N.A.® soil DNA Kit (Omega Bio-tek, Norcross, GA, USA). The primers used for bacterial 16S rRNA (V3-V4) gene amplifications were 338F (5′-ACTCCTACGGGAGGCAGCAG-3′) and 806R (5′-GGACTACHVGGGTWTCTAAT-3′) [29]. Amplifications of the internal transcribed spacer region ITS2 in fungi were conducted using the primers ITS3F (5′-GCATCGATGAAGAACGCAGC-3′) and ITS4R (5′-TCCTCCGCTTATTGATATGC3′) [30]. Before endophytic DNA extractions, leaves or roots were washed with sterile H_2_O and then treated with 70% ethanol for 5 min. After that, the samples were immersed in 5.25% NaClO solution for 5 min, and in 70% ethanol for 30 s. Finally, the samples were washed with sterile H_2_O [31]. A two-round amplification process was used to amplify the 16S rRNA amplicon of bacteria inhabiting roots and leaves. The first round of PCR was performed using the primers 799F (5′-AACMGGATTAGATACCCKG-3′) and 1392R (5′-ACGGGCGGTGTGTRC-3′) [31]. The second round of PCR was conducted with the primers 799F (5′-AACMGGATTAGATACCCKG-3′) and 1193R (5′-ACGTCATCCCCACCTTCC-3′) targeting the V5–V7 region [32]. The amplification of endophytic fungi was also performed using the ITS3F/ITS4R primers. All samples were sequenced on an Illumina MiSeq platform (Illumina, San Diego, USA) with a paired end sequencing (2 × 300). Low-quality read ends and primer sequences were trimmed. Paired end sequences were merged to a single sequence by FLASH version 1.2.11 [33]. Operational taxonomic units (OTUs) with 97% similarity cutoff were clustered using UPARSE version 7.1 [34] after removing chimeric sequences and singletons. Representative sequences were classified using RDP Classifier version 11.5 against the SILVA reference database (v. 132) and UNITE (v. 8.0) to classify each OTU into bacteria and fungi, respectively. The raw data of bacterial and fungal sequences are available at the National Center for Biotechnology Information under BioProject ID PRJNA797907.

### Physiological traits and transcriptome sequencing

Physiological traits of roots and young leaves, including pH, total phenols, tannin, NH_4_^+^, NO_3_^−^, and starch, were measured for four replicates per treatment. The remaining leaves and roots after DNA extraction were separated into two parts. One part was dried at 75 °C for 72 h and ground into fine powder, for which measurements of pH and starch were conducted. Mixtures of dried samples and deionized water (volume ratio 1: 8) were shaken at 250 rpm for 1 h and then centrifuged at 13,000 rpm for 5 min to test pH [35]. Fine powder was extracted by 80% ethanol (v/v) at 80 °C for 30 min, and the supernatant was removed. The residuals were hydrolyzed by 9.2 M HClO_4_, and an anthrone reagent was used to measure starch [8]. Fresh samples (the second part) of roots and young leaves were ground into powder to determine NH_4_^+^ and NO_3_^−^ [27]. Fresh roots or young leaves (0.5 g) were extracted using 95% ethanol for 2 h at 60 °C. A folin reagent and 20% Na_2_CO_3_ (g/L) were added into the extract for 0.5 h at 30 °C and then tested for total phenols at 760 nm.

To compare males and females, a transcriptome analysis of young leaves was performed between control- and limited-nitrogen treatments (N1 and N3). Three replicates per treatment were analyzed. Total RNA was extracted using Plant RNA Purification Reagent, and genomic DNA was removed using DNase I (TaKara). A detailed description of the RNA quality determination, library preparation, and Illumina Hiseq sequencing are available in Methods S2. The clean reads were separately aligned using the reference genome *Populus_trichocarpa* (http://plants.ensembl.org/Populus_trichocarpa/Info/Index). RSEM was used to quantify gene abundances (http://deweylab.biostat.wisc.edu/rsem/). The expression level of each transcript was calculated according to the transcript per million reads method. Heatmaps of the expression profiles were generated using the heatmap package in R. A differential expression analysis was performed to identify significantly differently expressed genes (DEGs) using DESeq2 (*P*-adjust value < 0.05, |log2FC|>1) [11]. A functional-enrichment analysis (KEGG) was performed to identify which DEGs were significantly enriched at the Bonferroni-corrected *P-*value <0.05 compared with the whole-transcriptome background.

### Statistical analysis

The effects of sex and N treatment on soil chemicals, plant physiological traits, and Shannon diversity were analyzed by a two-way ANOVA analysis. The beta diversity of fungi and bacteria was assessed using nonmetric multidimensional scaling (NMDS) based on Bray-Curtis distances. The significance of sex, N treatment, and their interaction effects on community dissimilarity was tested with PERMANOVA by using the “adonis” function in vegan package in R [36]. A hierarchical clustering analysis was performed by using the method “Ward.D2” with “hclust ()” function in R. The Venn diagram analysis was used to define bacterial genera and fungal OTUs shared or not shared among rhizosphere soil, roots, old leaves, and young leaves and visualized in Cytoscape (v. 3.5). Interactions and connections of physiological traits with the alpha diversity and dominant bacterial/fungal taxa were assessed by Mantel test with vegan package in R [37]. The co-occurrence networks were visualized in Gephi and performed by Conet in Cytoscape (v. 3.5) to show the network topological attributes of bacterial and fungal communities [11]. Only significant (*P* < 0.01) and robust Spearman’s correlations (*r* > 0.6 or *r* < 0.6) were kept in the networks. The linear discriminant analysis effect size (LEfSe) with logarithmic LDA> 3 (Wilcoxon *P* < 0.05) was applied to identify biomarker taxa of different niches to show the effects of sex. In each niche, the EdgeR’ was used to reveal different abundances of OTUs. OTUs present in at least 60% of samples and with a relative abundance ≥ 0.1% were defined as dominant taxa. The phylogenetic tree of roots and young leaves was annotated and visualized in iTOL software [11]. The dominant taxa, biomarker taxa (LEfSe), and hub nodes among networks were defined as keystone taxa.

## Results

**Fig. 1:**
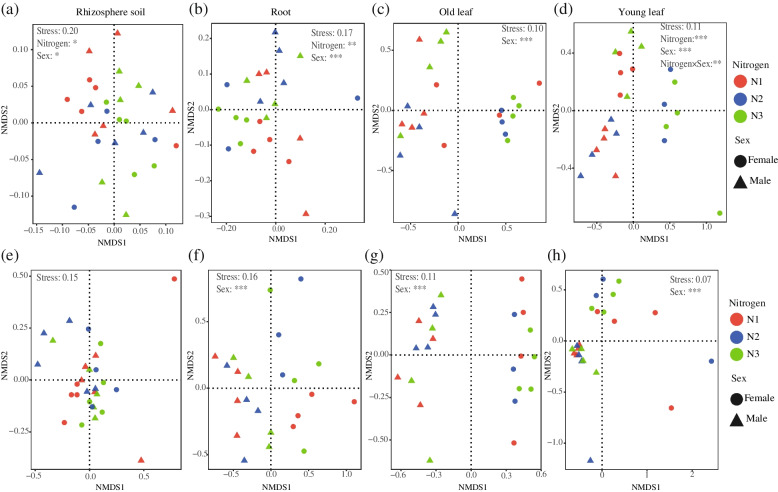
The impacts of sex and nitrogen (N) treatments on bacterial (**a**–**d**) and fungal (**e**–**h**) community structures based on nonmetric multidimensional scaling (NMDS) analysis. **a**, **e** Rhizosphere soil, **b**, **f** roots, **c**, **g** old leaves, **d**, **h** young leaves. N1: control-nitrogen treatment, N2: limited N re-irrigated with control-nitrogen solution treatment and N3: limited-nitrogen treatment. Three replicates of female samples (roots, and old and young leaves) at N2. PERMANOVA revealed sex, N treatment and their interaction effects on microbial communities

The differences of bacterial communities in the rhizosphere soil, roots, and young leaves were significantly affected by soil N (Fig. 1). However, sex imposes stronger effects on bacterial communities than soil N levels with clear differences between the roots, old leaves, and young leaves of males and females. The beta diversity of the bacterial community of old leaves was significantly affected only by sex (Fig. 1c). Similarly, sex was the main factor in driving differences of fungal communities in roots, old leaves, and young leaves (Fig. 1f–h).

**Fig. 2:**
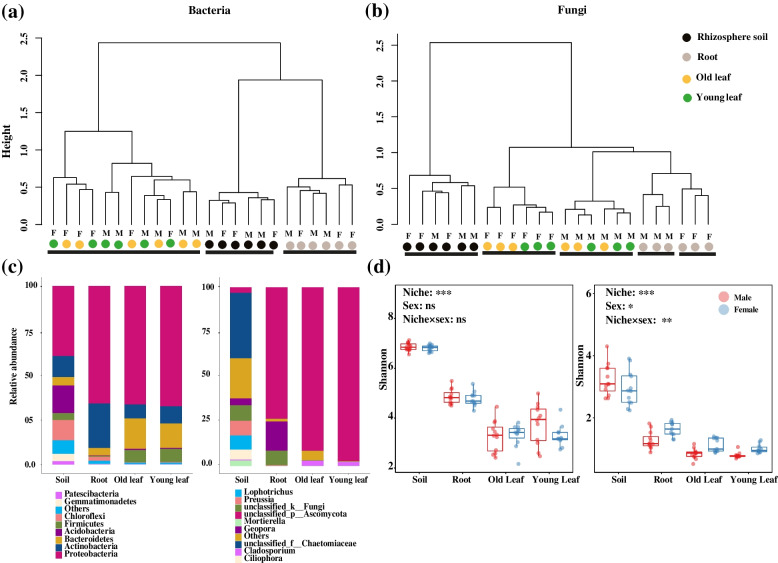
Strong niche differentiation shaping plant microbiomes. Hierarchical clustering analysis (**a**, **b**), relative abundance (**c**), and Shannon diversity (**d**) of bacterial and fungal communities in rhizosphere soil, roots, old leaves, and young leaves

The hierarchical clustering analysis revealed three clusters in bacterial communities: rhizosphere soil, roots, and leaves (old and young) were well separated at the genus level (Fig. 2a). The fungal communities of the rhizosphere soil, roots, and leaves (old and young) were well separated, as also the fungal communities of male and female roots (Fig. 2b). The relative abundance of Acidobacteria and Chloroflexi declined in roots, old leaves, and young leaves relative to the rhizosphere soil (Fig. 2c). Unclassified genera belonging to Ascomycota were enriched in roots, old leaves, and young leaves (Fig. 2c). The relative abundance of Actinobacteria and *Geopora* was significantly higher in roots than in other parts in different treatments (Fig. S1). The alpha diversity of bacterial and fungal communities was mainly affected by the niche, the rhizosphere soil showing the highest diversity (Fig. 2d). The alpha diversity of fungal communities was significantly impacted by sex as well (Fig. 2d, Fig. S2b).

**Fig. 3:**
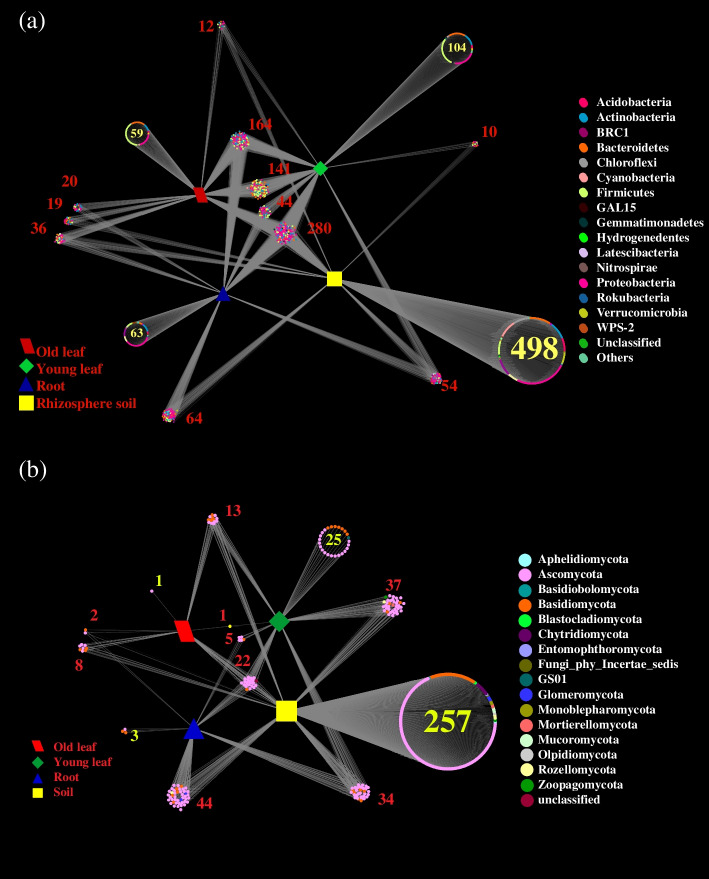
Venn diagrams showing shared and specific bacterial OTUs in different niches including rhizosphere soil, roots, old leaves, and young leaves. **a** Bacterial community, **b** fungal community. Analysis of bacterial and fungal communities were based on genus and OTU level, respectively

It was found that the four niches harbored 280 bacterial genera and 22 fungal OTUs in common, the majority of them belonging to Proteobacteria and Ascomycota (Fig. 3). The rhizosphere soil, roots, old leaves, and young leaves had 498, 63, 59, and 104 unique bacterial genera, and 257, 3, 1, and 25 unique fungal OTUs, respectively (Fig. 3). The numbers of bacterial genera belonging to Bacteroidetes and Firmicutes were much higher in old and young leaves than in roots and soil (Fig. 3a). The differential abundance analysis indicated that more fungal OTUs were absent in the endophytes of females compared to males (Fig. S3).

**Fig. 4:**
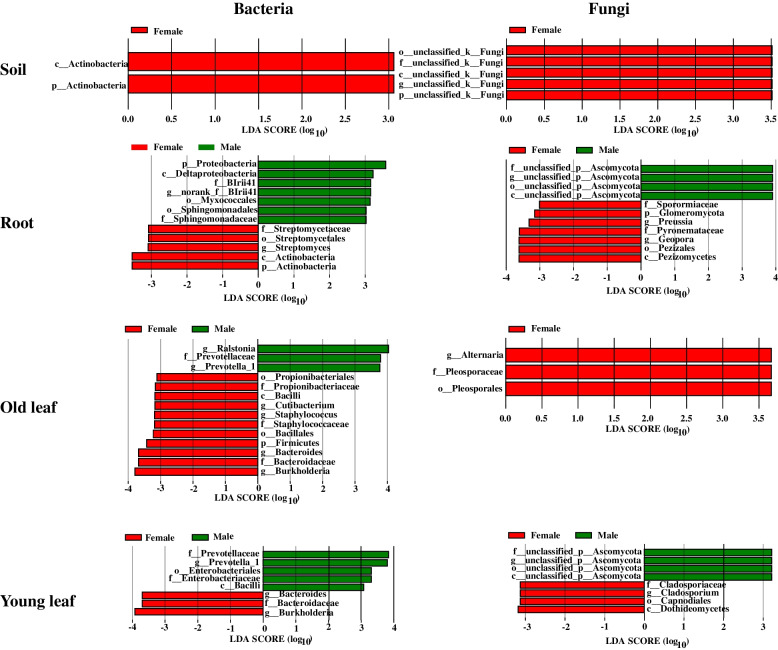
The identification of biomarker taxa affected by sex through linear discriminant analysis effect size (LEfSe) with logarithmic LDA> 3 (Wilcoxon *P* < 0.05). The left parallels indicated biomarker taxa of bacteria at each niche, while the right left parallels indicated biomarker taxa of fungi at each niche

The LDA effect size of LEfSe clearly indicated the presence of bacterial and fungal biomarker taxa in different niches (Fig. 4). The most significant bacterial biomarker taxon in the rhizosphere soil of *P. cathayana* females was Actinobacteria. The most significant bacterial and fungal biomarker taxa in the root endosphere of females were Actinobacteria and the genus *Geopora*, respectively, while in the root endosphere of males they were Proteobacteria and a genus belonging to Ascomycota, respectively. Other significant bacterial and fungal biomarker taxa were found in the old leaf endosphere. Young leaves of *P. cathayana* males and females also harbored distinct bacterial and fungal biomarker taxa.

**Fig. 5:**
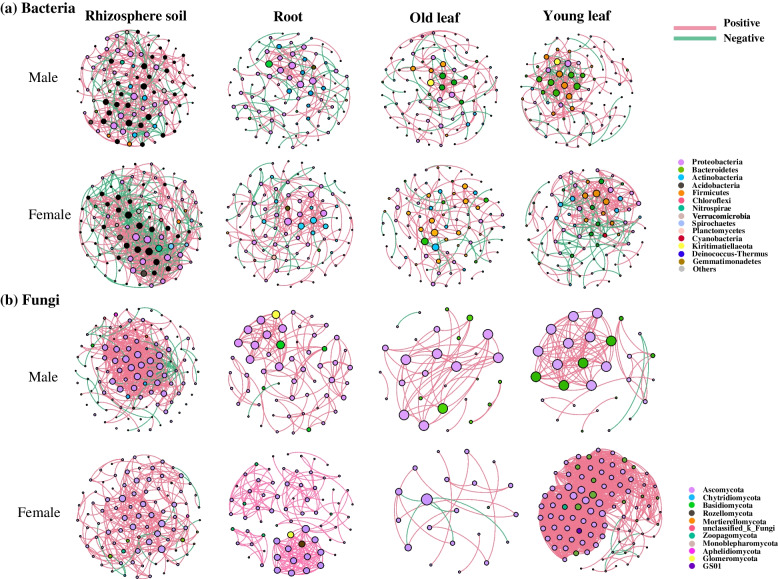
Different effects between *P. cathayana* males and females on the networks of bacterial (**a**) and fungal (**b**) communities in rhizosphere soil, roots, old leaves, and young leaves

To further characterize the niche and sex effect on plant microbiomes, particularly on plant endophytes, the co-occurrence patterns of bacterial and fungal communities were assessed along the soil-plant continuum. Bacterial communities of the rhizosphere soil and young leaves showed a higher network complexity than those of roots or old leaves with a higher average degree and clustering coefficient (Fig. S4a; Table S1). However, fungal communities of young leaves showed the highest network complexity with the highest average degree (40.25) and clustering coefficient (0.75) (Fig. S4b; Table S1). The bacterial taxonomic composition of “hub nodes” in the network differed between the rhizosphere soil and plant compartments (Fig. 5a). Positive edges and the average degree of bacterial networks were higher in the rhizosphere soil, roots, old leaves, and young leaves of females when compared to males, while positive edges and the average degree of fungal communities in young leaves of *P. cathayana* females were much higher than those of other niches (Table 1). OTUs belonging to Bacteroidetes and Firmicutes in young leaves of *P. cathayana* males, and OTUs belonging to Proteobacteria and Firmicutes in young leaves of females were more connected with others (Fig. 5b). OTUs belonging to Ascomycota in young female leaves were more connected with other OTUs in the network (Fig. 5b).

**Table 1:** Different effects of *P. cathayana* males and females on bacterial and fungal co-occurrence network characteristics

	Positive edge	Negative edge	Average degree	Modularity	Average clustering coefficient	Average path distance
**Bacteria**						
Soil						
Male	250	92	6.980	0.544	0.472	3.151
Female	353	202	11.443	0.394	0.556	2.663
Root						
Male	86	52	3.172	0.676	0.308	5.481
Female	142	34	3.745	0.626	0.408	4.452
Old leaf						
Male	93	27	2.759	0.677	0.315	6.380
Female	105	25	2.955	0.711	0.410	4.619
Young leaf						
Male	159	42	4.729	0.451	0.438	3.902
Female	163	69	5.659	0.493	0.381	4.385
**Fungi**						
Soil						
Male	431	48	9.124	0.308	0.450	2.961
Female	251	12	5.656	0.500	0.347	3.453
Root						
Male	118	1	4.250	0.783	0.877	2.419
Female	197	0	5.549	0.700	0.896	2.498
Old leaf						
Male	48	1	3.379	0.622	1.000	1.000
Female	15	5	1.905	0.765	0.902	1.259
Young leaf						
Male	92	2	6.714	0.299	0.900	1.021
Female	1246	6	27.516	0.339	0.823	2.205

**Fig. 6:**
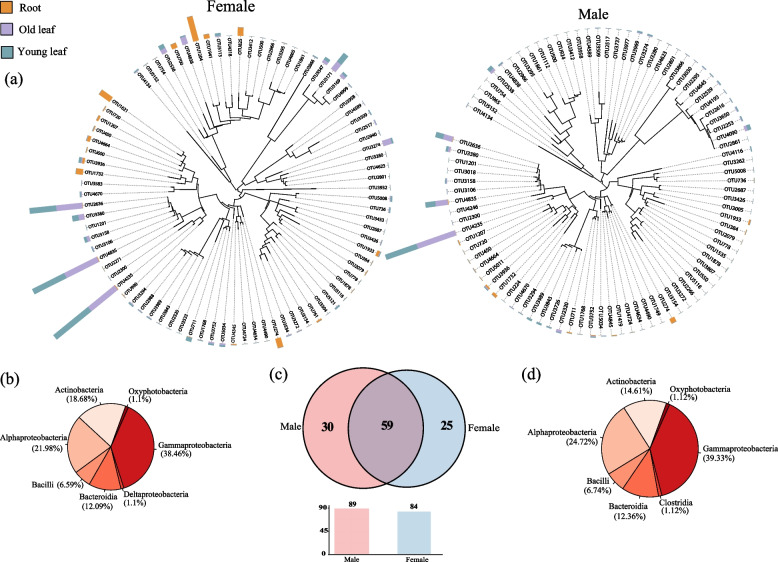
Phylogenetic tree, taxonomic composition, and distribution patterns of dominant bacterial taxa living in roots, old leaves, and young leaves. **a** The identification of dominant bacterial taxa in *P. cathayana* females and males, respectively. **b** The distribution pattern of dominant bacterial taxa in *P. cathayana* females. **c** The number of dominant bacterial taxa, the shared, and specific bacterial OTUs in *P. cathayana* females and males. **d** The distribution pattern of dominant bacterial taxa in *P. cathayana* males

In microbial communities of the endosphere, the dominant bacterial OTUs mainly belonged to Gammaproteobacteria, Alphaproteobacteria, and Actinobacteria at the class level, while dominant fungal OTUs were affiliated with Ascomycota at the genus level (Fig. 6, Fig. S5). A total of 114 and 20 dominant OTUs of bacteria and fungi, respectively, were identified. *P. cathayana* males and females harbored 30 and 25 distinct dominant bacterial OTUs, while 5 and 4 unique and dominant fungal OTUs, respectively. Unique dominant bacterial OTU1204, OTU1621, and OTU 825 in the female root endosphere showed a higher relative abundance than other root endophytes (Fig. 6a). The shared OTU4235, OTU4835, and OTU2362 between males and females had higher relative abundances than other leaf (both old and young) endophytes. Dominant fungal OTU229 and OTU125 with much higher relative abundances than other dominant OTUs were shared among roots, old leaves, and young leaves in both males and females (Fig. S5).

**Fig. 7:**
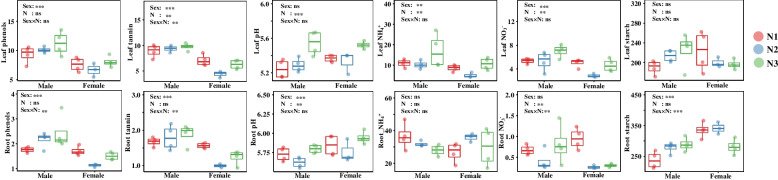
Effects of sex and N treatments on plant physiological traits. Two-way ANOVA analysis was performed. Sex: sex effect, N: N treatment effect, sex×N: sex and N interaction effect. ns: not significant, * 0.01 < *P* ≤ 0.05, ** 0.001 < *P* ≤ 0.01, *** *P* ≤ 0.001

Sex significantly affected soil total nitrogen, total phosphorus, NH_4_^+^, NO_3_^−^, microbial carbon, microbial nitrogen, and NAG (Table S2). Nitrogen and sex showed significant effects on plant growth (Table S3). Concentrations of phenols, tannins, NH_4_^+^ and NO_3_^−^ of young leaves, concentrations of phenols, tannin, and starch, and the pH level of roots were significantly affected by sex (Fig. 7). The soil N treatment also showed significant effects on tannins, pH, NH_4_^+^ and NO_3_^−^ in young leaves, and on pH and of NO_3_^−^ in roots (Fig. 7). Young male and female leaves showed great differences in gene expression, females being more sensitive to the N treatment, as revealed by transcriptomic data (Fig. S6). A total of 37,008 genes were identified. The gene expression analysis revealed 272 genes that were significantly upregulated in males, and 275 genes that were significantly downregulated in females in the control treatment. The N-limited treatment caused much greater differences in gene expression between males and females (Fig. S6b). The KEGG pathway enrichment showed that more genes related to plant-pathogen interaction functions were significantly different between males and females in control and N-limited conditions (Fig. S6c).

**Fig. 8:**
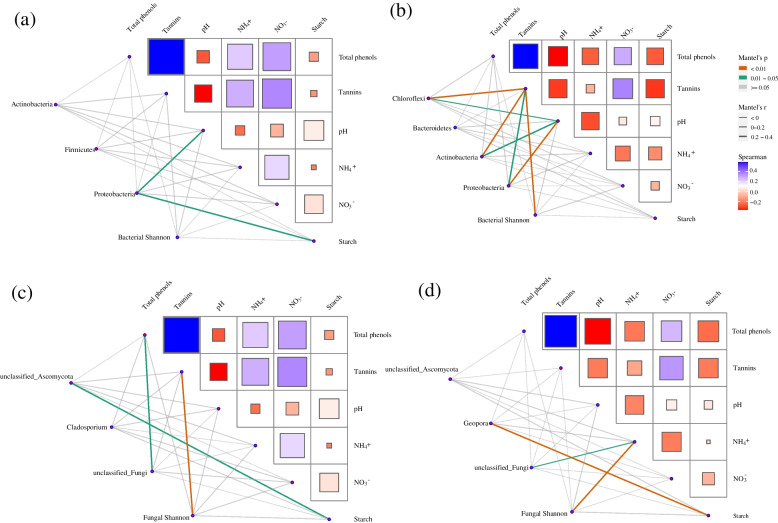
Correlations of physiological traits with abundant taxa and diversity of endophytes in roots and young leaves. NH_4_^+^, ammonium; NO_3_^−^, nitrate. **a**–**c** indicated young leaf, **b**, **d** indicated root

The pH value was negatively related with tannins in young leaves and roots (Fig. 8). Starch and pH of young leaves had significant effects on the abundance of Proteobacteria, while tannins and pH of roots showed significant effects on the abundance of Chloroflexi, Actinobacteria, and Proteobacteria, and bacterial Shannon diversity (Fig. 8a, b). The abundance of unclassified genera belonging to Ascomycota was significantly affected by starch, while the fungal Shannon diversity was affected by tannins in young leaves (Fig. 8c). The abundance of *Geopora* was affected by starch, while the fungal Shannon diversity was affected by the NH_4_^+^ concentration of roots (Fig. 8d).

## Discussion

Our initial hypothesis was that sex is an important factor influencing the properties (including microbial communities) and functions of the rhizosphere soil. To adapt to varied environments, different plant species or genotypes are able to recruit beneficial microbes and vary interactions among microbes in the rhizosphere soil through changing chemical conditions and by releasing root-derived compounds [13, 22, 38]. The contents of total nitrogen, total phosphorus, NH_4_^+^, and NO_3_^−^ were lower in the rhizosphere soil of females compared to males, probably because of higher nutrient demands of *P. cathayana* females for growth (Tables S2 and S3).

Different chemical traits of the rhizosphere soil have been reported as being important factors influencing bacterial and fungal communities and functions in *P. euphratica* males and females in natural forests [22]. Another important factor is the microbial substrate preferences to different root exudates [38], while the quality and amount of photosynthesis-derived exudates in *P. cathayana* males and females are likely to be different because of differences in carbon fixation and allocation, nutrient uptake, and utilization, and connections with arbuscular mycorrhizal fungi [7, 8, 26]. In this study, sex greatly altered interactions among hub nodes of different OTUs as indicated by the co-occurrence network, the biomarker taxa, and the microbial carbon biomass of the rhizosphere soil (Table 1, S2; Figs. 4 and 5). Microbial enzyme activities dynamically respond to alterations in the nutrient and substrate availability and they represent the biological metabolism of the soil microbiome [39]. Sex significantly affected the NAG activity related to chitin degradation (Table S2), which reflected variations in soil properties and microbial communities imposed by *P. cathayana* males and females.

Differences have been reported in poplars [9, 40, 41] and other species [10, 11, 23] in the plant microbiome among soil, root, and other microhabitats. This study also demonstrated a strong niche differentiation, such as a large number of unique taxa in the rhizosphere soil, a decreased alpha diversity, and varied abundance of major taxa along the soil-*P. cathayana* continuum. Extreme pH conditions impose stronger selective pressures for the survival and fitness of microbes, which suggests a stochastic process in neutral conditions but a deterministic assembly in acidic or alkaline pH conditions [42]. This study revealed a clear decreasing pH gradient from neutral (rhizosphere soil) to acidic conditions (leaf) (Table S1, Fig. 7), which demonstrated an ecological filter during community assembly processes along the soil-root-leaf continuum. For example, the rapid decrease of Basidiomycota within plant compartments, particularly in young leaves, was likely to be related with the decreasing pH because most Basidiomycota taxa perform better in higher pH conditions [43]. Fine-tuning and adaptation of the microbial assembly and stability, e.g., in roots and leaves, are clearly evident [9, 11, 23]. Besides different pH conditions, phenol and tannin levels of young leaves were higher than those of roots, which indicated that the microbes needed to have an ability to deal with defense chemicals. However, the sex impacts on the endophyte composition and abundance in each niche were not amplified by the great differences in the transcriptome and physiological traits between males and females under nitrogen-limited conditions (Fig. S1). Particularly, the fungal community inhabiting the plant endosphere stayed stable under different nitrogen treatments, which demonstrated a stronger niche-depended selection instead of external environmental effects.

A successful colonization of bacteria and fungi in roots and leaves requires that these microbes have the capacity to overcome the host’s immune system in different microhabitats [9–11]. Aerial plant parts impose much stronger selective pressures than belowground parts on the microbiome assembly [11, 44]. Indeed, the composition and structure of microbes residing in leaves (both old and young) were very different compared to those belowground, while young and old leaves had a similar microbe composition, relative abundance, and alpha and beta diversity (Figs. 2 and 6, S1, S5). Fungal taxa from the phylum Ascomycota enrich in live and senescent leaves of trees [45]. For most microbes in this study, young leaves did not impose a stronger inhibitory influence on the colonization or growth of the endophytes than old leaves. It was evident that these bacterial and fungal microbes colonize leaves shortly after emergence and reach and keep a constant level before leaf abscission. However, some fungal taxa with a relatively low abundance (classified as “others” in this study) successfully colonized old leaves (Fig. S1b), probably as a consequence of the declined action of protective mechanisms resulting in a rapid proliferation of opportunistic fungi during leaf senescence [45, 46].

One interesting finding was that the network hubs in young leaves were more connected with other taxa in the fungal community than those in old leaves (Table 1; Fig. 5). Keystone species belonging to Basidiomycota were more connected with other taxa in young leaves than in old leaves (Fig. 5). Positive edges in the fungal network greatly declined from young to old leaves, implying substantially decreased competition among fungal species [11, 47]. A loosely assembled fungal network reflects subtle changes in the host status; old leaves are suggested to decompose under saprophytic fungi from Ascomycota before abscission [45, 48].

Our results further confirmed that the selective pressure imposed by sex on endophytes in different plant parts is much stronger than that on microbial communities of the rhizosphere soil. The community structure, biomarker/core taxa, and interactions among bacterial and fungal taxa in roots, old leaves, and young leaves were different between *P. cathayana* males and females. Key microbial taxa typically enriched in different parts of *P. cathayana* males and females, such as fast-growing Alphaproteobacteria and Gammaproteobacteria (Fig. 6), reflecting adaptation of these plant-associated microbes to interior environments of plants by acquiring carbohydrates and nutrients from the host [25]. For example, the Gammaproteobacteria species are capable of colonizing and then dominating diverse niches, including rhizosphere, and root and leaf endosphere, with crucial roles in regulating host fitness, pathogen suppression, and tolerance to varied stresses [49, 50]. However, different relative abundances of these taxa and distinct taxa in each plant compartment indicated sex specificity in the structure and function of entophytic communities (Fig. 6). *Streptomycetes* and *Geopora* were biomarker taxa and enriched in the female root endosphere (Fig. 4). *Streptomycetes* species colonizing root systems are considered beneficial due to their capacity to promote plant growth and induce defense responses against plant pathogens [51, 52]. *Geopora* had a higher relative abundance in the root endosphere than in the rhizosphere soil (Fig. 2), and the enrichment was greater in female roots (Fig. S1b).

Based on the limited knowledge on how male and female plants, especially in *Populus* species, resist biotic stresses, the enriched *Streptomycetes* species have the potential ability to enhance pathogen resistance in females. The species of the genus *Geopora* are important mycobionts of ectomycorrhizal fungi [53, 54]. Female roots probably establish a closer mutualistic relationship with *Geopora* species than male roots, which contributes to meeting the higher nitrogen demand of *P. cathayana* females during growth [8]. *Alternaria* species are mainly saprophytic fungi [55]. In this study, they were identified as biomarker taxa by LEfSe and also as dominant taxa in old leaves of *P. cathayana* females, indicating sex-specific differences in recruiting microbes related with the decomposition process before leaf abscission.

This study further found distinct bacterial and fungal biomarker and dominant taxa in young leaves of *P. cathayana* males and females (Fig. 4, S5). Interestingly, the fungal co-occurrence network was much more complex and the alpha diversity of young female leaves was significantly higher compared to male leaves (Fig. 5b, S2). The more connected network among OTUs from Ascomycota, Basidiomycota, Zoopagomycota, and other taxa suggested a closer relationship between female leaves and its associated microbes, which implied that the fitness of the female host was more dependent on the fungal community compared to males. Hub species in microbial networks are recognized as mediators between the host and its associated microbiome. Through network hubs, a host can selectively influence the assembly of their associated microbiome by regulating microbe-microbe interactions [12]. Variations in the constituting microbial taxa and in the number of hub microorganisms have a significant influence on the assembly of microbial communities, and this effect is suggested to be independent from external environmental factors [56].

Furthermore, the N levels in this study clearly caused sexually different gene expressions in various functions by transcriptomic data (Fig. S6). Previous studies also reported greatly differences in gene expressions and physiological traits at similar N gradient [20, 26, 27]. In the present study, substantial differences between males and females in gene expression related to plant-pathogen interactions were found (Fig. S6). However, these differences did not lead to great changes in the composition and structure of leaf endophyte (Figs. S1 and S2). The composition and abundance of endophytic communities in the re-irrigated nitrogen treatment were similar to those of other two N treatments. Instead, there was convincing evidence that a stronger control by a host rather than by the external N dose shapes specific endophytic communities, probably due to different traits in males and females. Plant-associated microbes differ in their ability to successfully colonize different plant parts due to their differences in overcoming cell wall, in resisting defense-related chemicals and in carbohydrate metabolic functions of the host [25, 57, 58]. Leaves of *P. cathayana* males have a higher mesophyll palisade tissue thickness but a lower spongy cell density [59], while males have a smaller root system compared to females [7]. These differences in the structure are probably important forces in selecting endophytes inhabiting leaves or roots in *P. cathayana* males and females.

Physiological traits of roots and young leaves were used to reflect the environment of the plant endosphere, and most of these traits were significantly affected by sex (Fig. 7). Leaf carbon is suggested being the main driving force for changes in the diversity, richness, and composition of foliar fungal endophytes [60], while many studies have emphasized that secondary metabolites of plants, particularly those related to plant defense traits, influence the plant microbiome [46]. However, after exploring twelve *P. trichocarpa* genotypes, which produced different amounts of salicylic acid-related secondary metabolites, Veach et al. [58] suggested that plant defense strategies partially drive colonization and the assembly of taxon-specific microorganisms. In addition, increased primary metabolites facilitate the colonization of suitable microbes within roots [61]. Our results were unable to explain the specific biological mechanisms that control a host plant’s microbiome. However, this study demonstrated that the microbial assembly was affected or driven by diverse conditions within plant tissues, such as pH, carbon supply (starch), nitrogen status (NH_4_^+^), or defense traits (total phenols and tannins) (Fig. 8). Previous empirical evidence has showed that plant hosts exert strong effects on plant microbiomes by deterministic selection during plant growth and development [11]. We found that *P. cathayana* males and females have sex-specific selection pressures or specialized endosphere habitats that microbes select to form distinct microbial compositions.

## Conclusions

In this study, bacterial and fungal community assemblies across a plant-soil continuum were mainly influenced by niches, which suggested that ecological filters, for example, a decreasing pH gradient from the rhizosphere soil to leaves, affect the community assembly processes along the soil-root-leaf continuum. Endophytic fungal communities showed a higher stability than bacterial communities in each niche in response to different soil N treatments, which demonstrated the presence of a powerful control of a host in selecting fungi. Our results suggested potentially different functions in N-cycling, nutrient acquisition, and pathogen resistance due to divergent microbial communities in the rhizosphere soil, roots, and leaves. All results provided important new knowledge for understanding the effects of sexual dimorphism in microbial assemblies and plant-microbiome interactions.

## Supplementary Information

**Additional file 1: **Supplementary methods. **Table S1.** Bacterial and fungal co-occurrence network characteristics in each niche. **Table S2.** The physiological traits and microbial functions of rhizosphere soil affected by sex and nitrogen treatment. **Table S3.** The growth characteristics of *P. cathayana* males and females under different N treatments. **Figure S1.** The composition and relative abundance of bacterial (a) and fungal (b) communities in the rhizosphere soil, roots, old leaves and young leaves of *P. cathayana* males and females in different N treatments. **Figure S2.** The Shannon diversity of bacterial and fungal communities in different N treatments. From left to right: rhizosphere soil, roots, old leaves and young leaves. **Figure S3.** The volcano plot illustrating the enrichment and depletion patterns of bacterial and fungal microbiomes in each niche (male vs female). **Figure S4.** Different niche effects on the networks of bacterial (a) and fungal (b) communities. **Figure S5.** Phylogenetic tree, taxonomic composition, and distribution patterns of dominant fungal taxa living in roots, old leaves and young leaves. (a) Dominant fungal taxa in *P. cathayana* females and males, respectively. (b) The distribution pattern of dominant fungal taxa in *P. cathayana* females. (c) The number of dominant fungal taxa, the shared and specific bacterial OTUs in *P. cathayana* females and males. (d) The distribution pattern of dominant fungal taxa in *P. cathayana* males. **Figure S6.** Heatmaps of the expression profiles (a), differential expression analysis (DEGs) (b) and functional-enrichment analysis (KEGG) (c). MC, young male leaves in control-nitrogen treatment; MN, young male leaves in limited-nitrogen treatment; FC, young female leaves in control-nitrogen treatment; FN, young female leaves in limited-nitrogen treatment.

## Data Availability

All bacterial and fungal databases in this study are available at National Center for Biotechnology Information under BioProject ID PRJNA797907.

## Abbreviations

NMDS: Nonmetric multidimensional scaling
TN: Total nitrogen concentration
AP: Available phosphorus concentration
TP: Total phosphorus concentration
LEfSe: Linear discriminant analysis effect size
NAG: β-1,4-N-acetylglucosaminidase activity

## References

[1] Renner SS (2014). The relative and absolute frequencies of angiosperm sexual systems: dioecy, monoecy, gynodioecy, and an updated online database. Am J Bot.

[2] Xue L, Wu H, Chen Y, Li X, Yin T (2020). Evidences for a role of two Y-specific genes in sex determination in *Populus deltoides*. Nat Commun.

[3] Bertiller MB, Sain CL, Bisigato AJ, Coronato FR, Aries JO, Graff P (2002). Spatial sex segregation in the dioecious grass Poa ligularis in northern Patagonia: the role of environmental patchiness. Biodivers Conserv.

[4] Hultine KR, Grady KC, Wood TE, Shuster SM, Stella JC, Whitham TG (2016). Climate change perils for dioecious plant species. Nat Plants.

[5] Liu M, Korpelainen H, Li CY (2021). Sexual differences and sex ratios of dioecious plants under stressful environments. J Plant Ecol.

[6] Avila-Sakar G, Romanow CA (2012). Divergence in defence against herbivores between males and females of dioecious plant species. Int J Evol Biol.

[7] Xia ZC, He Y, Yu L, Lv RB, Korpelainen H, Li CY (2020). Sex-specific strategies of phosphorus (P) acquisition in *Populus cathayana* as affected by soil P availability and distribution. New Phytol.

[8] Wu XY, Liu JT, Meng QQ, Fang SY, Kang JY, Guo QX (2021). Differences in carbon and nitrogen metabolism between male and female *Populus cathayana* in response to deficient nitrogen. Tree Physiol.

[9] Beckers B, Beeck M, Weyens N, Boerjan W, Vangronsveld J (2017). Structural variability and niche differentiation in the rhizosphere and endosphere bacterial microbiome of field-grown poplar trees. Microbiome.

[10] Bernard J, Wall CB, Costantini MS, Rollins RL, Amend AS (2021). Plant part and a steep environmental gradient predict plant microbial composition in a tropical watershed. ISME J.

[11] Xiong C, Singh BK, He JZ, Han YL, Li PP, Wan LH (2021). Plant developmental stage drives the differentiation in ecological role of the maize microbiome. Microbiome.

[12] Trivedi P, Leach JE, Tringe SG, Sa TM, Singh BK (2020). Plant-microbiome interactions: from community assembly to plant health. Nat Rev Microbiol.

[13] Guo QX, Yan LJ, Korpelainen H, Niinemets Ü, Li CY (2019). Plant-plant interactions and N fertilization shape soil bacterial and fungal communities. Soil Biol Biochem.

[14] Vega-Frutis R, Munguía-Rosas MA, Varga S, Kytöviita MM (2013). Sexspecific patterns of antagonistic and mutualistic biotic interactions in dioecious and gynodioecious plants. Perspec Plant Ecol Evol Syst.

[15] Wu QP, Tang Y, Dong TF, Liao YM, Li DD, He XH, Xu X (2018). Additional AM fungi inoculation increase *Populus cathayana* intersexual competition. Front Plant Sci.

[16] Stringlis IA, Yu K, Feussner K, Jonge RD, Bentum SV (2018). MYB72-dependent coumarin exudation shapes root microbiome assembly to promote plant health. Proc Natl Acad Sci USA.

[17] He Y, Zhu ZD, Guo QX, Xia ZC (2021). Sex-specific interactions affect foliar defense compound accumulation and resistance to herbivores in *Populus cathayana*. Sci Total Environ.

[18] Randriamanana TR, Nybakken L, Lavola A, Aphalo PJ, Nissinen K, Julkunen-Tiitto R (2014). Sex-related differences in growth and carbon allocation to defence in *Populus tremula* as explained by current plant defense theories. Tree Physiol.

[19] Vega-Frutis R, Luna-Esquivel G, Figueroa-Esquivel EM (2018). Land-use change impact on mycorrhizal symbiosis in female and male plants of wild *Carica papaya* (Caricaceae). Symbiosis.

[20] Song HF, Cai ZY, Liao J, Tang DT, Zhang S (2019). Sexually differential gene expressions in poplar roots in response to nitrogen deficiency. Tree Physiol.

[21] Mushinski RM, Payne ZC, Raff JD, Craig ME, Pusede SE, Rusch DB (2020). Nitrogen cycling microbiomes are structured by plant mycorrhizal associations with consequences for nitrogen oxide fluxes in forests. Global Change Biol.

[22] Guo QX, Liu JT, Yu L, Korpelainen H, Li CY (2021). Different sexual impacts of dioecious *Populus euphratica* on microbial communities and nitrogen cycle processes in natural forests. Forest Ecol Manage.

[23] Hou SJ, Thiergart T, Vannier N, Mesny F, Ziegler J, Pickel B, Hacquard S (2021). A microbiota-root-shoot circuit favours Arabidopsis growth over defence under suboptimal light. Nat Plants.

[24] Lebeis SL, Paredes SH, Lundberg DS, Breakfield N, Gehring J, McDonald M (2015). Salicylic acid modulates colonization of the root microbiome by specific bacterial taxa. Science.

[25] Cole BJ, Feltcher ME, Waters RJ, Wetmore KM, Visel A (2018). Genome-wide identification of bacterial plant colonization genes. PLoS Biol.

[26] Zhang S, Jiang H, Zhao HX, Korpelainen H, Li CY (2014). Sexually different physiological responses of *Populus cathayana* to nitrogen and phosphorus deficiencies. Tree Physiol.

[27] Luo J, Zhou J, Li H, Shi W, Polle A, Lu M, Sun X, Luo ZB (2015). Global poplar root and leaf transcriptomes reveal links between growth and stress responses under nitrogen starvation and excess. Tree Physiol.

[28] Schindlbacher A, Rodler A, Kuffner M, Kitzler B, Sessitsch A, Zechmeister-Boltenstern S (2011). Experimental warming effects on the microbial community of a temperate mountain forest soil. Soil Biol Biochem.

[29] Caporaso JG, Lauber CL, Walters WA, Berglyons D, Lozupone CA, Turnbaugh PJ, Fierer N, Knight R (2011). Global patterns of 16S rRNA diversity at a depth of millions of sequences per sample. Proc Natl Acad Sci USA.

[30] White TJ, Bruns TD, Lee S, Taylor J, Innis MA, Gelfaud DH, Sninsky JJ, White TJ (1990). Amplification and direct sequencing of fungal ribosomal RNA genes for phylogenetics. PCR protocols: a guide to methods and applications.

[31] Samad A, Trognitz F, Compant S, Antonielli L, Sessitsch A (2017). Shared and host-specific microbiome diversity and functioning of grapevine and accompanying weed plants. Environ Microbiol.

[32] Wang F, Men X, Zhang G, Liang KC, Xin YH, Wang J, Li AJ, Zhang HB, Liu HB, Wu LJ (2018). Assessment of 16S rRNA gene primers for studying bacterial community structure and function of aging flue-cured tobaccos. AMB Expr.

[33] Magoč T, Salzberg SL (2011). FLASH: fast length adjustment of short reads to improve genome assemblies. Bioinformatics.

[34] Edgar RC (2013). UPARSE: highly accurate OTU sequences from microbial amplicon reads. Nat Methods.

[35] Cornelissen J, Quested H, van Logtestijn R, Pérez-Harguindeguy M, Gwynn-Jones D, Díaz S, Callaghan TV, Press MC, Aerts R (2006). Foliar pH as a new plant trait: can it explain variation in foliar chemistry and carbon cycling processes among subarctic plant species and types?. Oecologia.

[36] Oksanen J, Kindt R, Legendre P, O’Hara B, Stevens MHH, Oksanen MJ, Suggests M (2007). The vegan package. Community Ecol Package.

[37] Sunagawa S, Coelho LP, Chaffron S (2015). Structure and function of the global ocean microbiome. Science.

[38] Zhalnina K, Louie KB, Hao Z, Mansoori N, da Rocha UN, Shi SJ (2018). Dynamic root exudate chemistry and microbial substrate preferences drive patterns in rhizosphere microbial community assembly. Nat Microbiol.

[39] Chen Y, Liu X, Hou YH, Zhou SR, Zhu B (2021). Particulate organic carbon is more vulnerable to nitrogen addition than mineral-associated organic carbon in soil of an alpine meadow. Plant Soil.

[40] Cregger MA, Veach AM, Yang ZK, Crouch MJ, Vilgalys R, Tuskan AG (2018). The *Populus* holobiont: dissecting the effects of plant niches and genotype on the microbiome. Microbiome.

[41] Bonito G, Reynolds H, Robeson MS, Nelson J, Hodkinson BP, Tuskan G, Schadt CW, Vilgalys R (2014). Plant host and soil origin influence fungal and bacterial assemblages in the roots of woody plants. Mol Ecol.

[42] Tripathi BM, Stegen JC, Kim M, Dong K, Adams JM, Lee YK (2018). Soil pH mediates the balance between stochastic and deterministic assembly of bacteria. ISME J.

[43] Xu F, Cai TJ, Yang X, Sui WZ (2017). Soil fungal community variation by large-scale reclamation in Sanjiang plain, China. Ann Microbiol.

[44] Wagner M, Lundberg D, del Rio T, Tringe SG, Dangl JL, Mitchell-Olds T (2016). Host genotype and age shape the leaf and root microbiomes of a wild perennial plant. Nat Commun.

[45] Voříšková J, Baldrian P (2013). Fungal community on decomposing leaf litter undergoes rapid successional changes. ISME J.

[46] Pang ZQ, Chen J, Wang TH, Gao CS, Li ZM, Guo LT, Xu JP, Cheng Y (2021). Linking plant secondary metabolites and plant microbiomes: a review. Front Plant Sci.

[47] van der Heijden MGA, Hartmann M (2016). Networking in the plant microbiome. PLoS Biol.

[48] Guerreiro MA, Brachmann A, Begerow D, Peroh D (2017). Transient leaf endophytes are the most active fungi in 1-year-old beech leaf litter. Fungal Divers.

[49] Mendes R, Kruijt M, de Bruijn I, Dekkers E, van der Voort M, Schneider JH, Piceno YM, DeSantis TZ, Andersen GL, Bakker PA, Raaijmakers JM (2011). Deciphering the rhizosphere microbiome for disease-suppressive bacteria. Science.

[50] Alvarez-Perez JM, Gonzalez-Garcia S, Cobos R, Olego MA, Ibanez A, Diez-Galan A, Garzon-Jimeno E, Coque JJR (2017). Use of endophytic and rhizosphere Actinobacteria from grapevine plants to reduce nursery fungal graft infections that lead to young grapevine decline. Appl Environ Microb.

[51] Salla TD, Thanise R, Astarita LV, Santarém ER (2014). Streptomyces rhizobacteria modulate the secondary metabolism of Eucalyptus plants. Plant Physiol Biochem.

[52] Tian X, Cao L, Tan H, Han W, Chen M, Liu Y, Zhou S (2007). Diversity of cultivated and uncultivated actinobacterial endophytes in the stems and roots of rice. Microb Ecol.

[53] Patterson A, Flores-Rentería L, Whipple A, Whitham T, Gehring C (2019). Common garden experiments disentangle plant genetic and environmental contributions to ectomycorrhizal fungal community structure. New Phytol.

[54] Shemesh H, Boaz BE, Millar CI, Bruns TD (2019). Symbiotic interactions above treeline of long-lived pines: Mycorrhizal advantage of limber pine (*Pinus flexilis*) over Great Basin bristlecone pine (*Pinus longaeva*) at the seedling stage. J Ecol.

[55] Jiang H, Shi YT, Zhou ZX, Yang C, Chen YJ, Chen LM, Yang MZ, Zhang HB (2011). Leaf chemistry and co-occurring species interactions affecting the endophytic fungal composition of Eupatorium adenophorum. Ann Microbiol.

[56] Agler MT, Ruhe J, Kroll S, Morhenn C, Kim ST, Weigel D, Kemen EM (2016). Microbial hub taxa link host and abiotic factors to plant microbiome variation. PLoS Biol.

[57] Levy A, Salas Gonzalez I, Mittelviefhaus M, Clingenpeel S, Paredes SH, Miao JM, Wang KR, Devescovi G, Stillman K, Monteiro F, Alvarez BR, Lundberg DS, Lu TY, Lebeis S, Jin Z, McDonald M, Klein AP, Feltcher ME, Rio TG, Grant SR, Doty SL, Ley RE, Zhao BY, Venturi V, Pelletier DA, Vorholt JA, Tringe SG, Woyke T, Dangl J (2018). Genomic features of bacterial adaptation to plants. Nat Genet.

[58] Veach AM, Morris R, Yip DZ, Yang ZK, Engle NL, Cregger MA (2019). Rhizosphere microbiomes diverge among *Populus trichocarpa* plant-host genotypes and chemotypes, but it depends on soil origin. Microbiome.

[59] Liu M, Liu XC, Du XH, Korpelainen H, Niinemets U, Li CY (2021). Anatomical variation of mesophyll conductance due to salt stress in *Populus cathayana* females and males growing under different inorganic nitrogen sources. Tree Physiol.

[60] Yang T, Weisenhorn P, Gilbert JA, Ni Y, Sun R, Shi Y, Chu H (2016). Carbon constrains fungal endophyte assemblages along the timberline. Environ Microbiol.

[61] Mangeot-Peter L, Tschaplinski TJ, Engle NL, Veneault-Fourrey C, Martin F, Deveau A (2020). Impacts of soil microbiome variations on root colonization by fungi and bacteria and on the metabolome of *Populus tremula* × *alba*. Phytobiomes J.

