# Supplementary material for: Plant sex affects plant-microbiome assemblies of dioecious Populus cathayana trees under different soil nitrogen conditions
Source: Microbiome. 2022 Nov 5;10:191. doi: 10.1186/s40168-022-01387-9 (PMC9636617; doi:10.1186/s40168-022-01387-9)

**Supplementary methods**

**Methods S1**

Control-nitrogen treatment: 0.5 mM KCl, 0.9 mM CaCl_2_, 0.3 mM MgSO_4_, 0.6 mM KH_2_PO_4_, 42 µM K_2_HPO_4_, 10 µM Fe-EDTA, 2 µM MnSO_4_, 10 µM H_3_BO_3_, 7 µM Na_2_MoO_4_, 0.05 µM CoSO_4_, 0.2 µM ZnSO_4_, 0.2 µM CuSO_4_, and 2 mM NH_4_NO_3_, pH 5.5.

Limited-nitrogen treatment: 0.5 mM KCl, 0.9 mM CaCl_2_, 0.3 mM MgSO_4_, 0.6 mM KH_2_PO_4_, 42 µM K_2_HPO_4_, 10 µM Fe-EDTA, 2 µM MnSO_4_, 10 µM H_3_BO_3_, 7 µM Na_2_MoO_4_, 0.05 µM CoSO_4_, 0.2 µM ZnSO_4_, and 0.2 µM CuSO_4_, pH 5.5.

**Methods S2**

1. RNA extraction

Total RNA was extracted from the tissue using Plant RNA Purification Reagent according to the manufacturer’s instructions (Invitrogen), and genomic DNA was removed using DNase I (TaKara). Then, RNA quality was determined by 2100 Bioanalyser (Agilent) and quantified using ND-2000 (NanoDrop Technologies). Only high-quality RNA samples (OD260/280=1.8~2.2, OD260/230≥2.0, RIN≥6.5) were used to construct sequencing library.

2. Library preparation, and Illumina Hiseq Sequencing

RNA-seq transcriptome library was prepared following the TruSeqTM RNA sample preparation Kit from Illumina (San Diego, CA) using 1 μg of total RNA. Shortly, messenger RNA was isolated according to the polyA selection method by oligo (dT) beads and then fragmented using the fragmentation buffer. Secondly, double-stranded cDNA was synthesized using a SuperScript double-stranded cDNA synthesis kit (Invitrogen, CA) with random hexamer primers (Illumina). Then, the synthesized cDNA was subjected to end-repair, phosphorylation and ‘A’ base addition according to the Illumina’s library construction protocol. Libraries were size-selected for cDNA target fragments of 300 bp on 2% Low Range Ultra Agarose, followed by PCR amplification using Phusion DNA polymerase (NEB) for 15 PCR cycles. After being quantified by TBS380, the paired-end RNA-seq sequencing library was sequenced with the Illumina HiSeq sequencer (2 × 150bp read length).

3. Read mapping

The raw paired-end reads were trimmed and quality-controlled by SeqPrep (https://github.com/jstjohn/SeqPrep) and Sickle (https://github.com/najoshi/sickle) with default parameters. Then, clean reads were separately aligned to *Populus_trichocarpa* (http://plants.ensembl.org/Populus_trichocarpa/Info/Index). The mapped reads of each sample were assembled by StringTie.

**Supplementary tables**

**Table S1** Bacterial and fungal co-occurrence network characteristics in each niche.

| **Bacteria** | Positive edge | Negative edge | Average degree | Modularity | Average clustering coefficient | Average path distance |
| --- | --- | --- | --- | --- | --- | --- |
| Niche |  |  |  |  |  |  |
| Soil | 494 | 231 | 14.646 | 0.382 | 0.495 | 2.322 |
| Root | 254 | 87 | 7.031 | 0.453 | 0.367 | 2.927 |
| Old leaf | 342 | 181 | 11.370 | 0.284 | 0.460 | 3.398 |
| Young leaf | 309 | 144 | 10.295 | 0.367 | 0.505 | 3.215 |
| **Fungi** |  |  |  |  |  |  |
| Niche |  |  |  |  |  |  |
| Soil | 540 | 15 | 11.935 | 0.245 | 0.468 | 2.733 |
| Root | 210 | 4 | 4.553 | 0.731 | 0.571 | 5.068 |
| Old leaf | 64 | 7 | 3.463 | 0.653 | 0.632 | 4.000 |
| Young leaf | 1966 | 6 | 40.245 | 0.148 | 0.749 | 1.845 |

**Table S2** The physiological traits and microbial functions of rhizosphere soil affected by sex and nitrogen treatment.

|  |  | **pH** | **TN** | **TP** | **AP** | **NH_4_^+^** | **NO_3_^-^** | **Microbial C** | **Microbial N** | **BG** | **NAG** |
| --- | --- | --- | --- | --- | --- | --- | --- | --- | --- | --- | --- |
| **Male** | N1 | 7.97±0.11 | 0.65±0.01 | 0.81±0.03 | 32.08±2.14 | 3.68±0.25 | 308.84±20.53 | 141.92±7.26 | 18.29±2.10 | 1.56±0.02 | 2.54±0.04 |
|  | N2 | 7.59±0.14 | 0.65±0.04 | 0.74±0.06 | 42.26±2.34 | 4.01±0.23 | 327.22±31.93 | 140.48±6.59 | 21.91±5.43 | 1.49±0.06 | 1.51±0.10 |
|  | N3 | 7.86±0.09 | 0.64±0.06 | 0.72±0.03 | 45.11±2.81 | 4.59±0.35 | 266.10±16.03 | 175.17±17.12 | 25.59±2.82 | 1.40±0.04 | 1.43±0.06 |
| **Female** | N1 | 7.72±0.08 | 0.53±0.03 | 0.63±0.05 | 35.43±2.47 | 3.91±0.27 | 214.32±23.30 | 125.53±7.82 | 21.86±1.02 | 1.46±0.02 | 1.55±0.08 |
|  | N2 | 7.80±0.08 | 0.55±0.04 | 0.66±0.02 | 39.00±1.90 | 3.37±0.31 | 250.68±30.61 | 119.79±5.72 | 26.86±3.98 | 1.47±0.04 | 1.46±0.07 |
|  | N3 | 7.89±0.06 | 0.53±0.05 | 0.60±0.04 | 38.85±1.49 | 3.40±0.19 | 237.87±33.81 | 114.56±5.87 | 22.67±1.49 | 1.42±0.06 | 1.58±0.11 |
| ***P* value** | Sex | ns | ** | ** | ns | * | ** | *** | ns | ns | *** |
|  | N | ns | ns | ns | ** | ns | ns | ns | ns | ns | *** |
|  | Sex×N | ns | ns | ns | ns | * | ns | ns | ns | ns | *** |

TN, total nitrogen (g·kg^-1^); TP, total phosphorus (g·kg^-1^); AP, available phosphorus (mg·kg^-1^); NH_4_^+^, ammonium (mg·kg^-1^); NO_3_^-^, nitrate (mg·kg^-1^); microbial C, soil microbial carbon biomass (mg·kg^-1^);microbial N, soil microbial nitrogen biomass (mg·kg^-1^); BG, β-1,4-glucosidase (μmol·g^-1^ min^-1^); NAG, β-1,4-N-acetylglucosaminidase (μmol·g^-1^ min^-1^). Two-way ANOVA analysis was performed. Sex: sex effect, N: N treatment effect, sex×N: sex and N interaction effect. ns: not significant, * 0.01 < *P* ≤ 0.05, ** 0.001 < *P* ≤ 0.01, *** *P* ≤ 0.001. N1: control-nitrogen treatment, N2: limited N re-irrigated with control-nitrogen solution treatment, and N3: limited-nitrogen treatment.

**Table S3** The growth characteristics of *P. cathayana* males and females under different N treatments.

| Sex | N treatments | Leaf (g) | Stem (g) | Root (g) | Total (g) |
| --- | --- | --- | --- | --- | --- |
| **Male** | N1 | 4.40±0.42 | 7.99±1.05 | 2.64±0.18 | 15.03±1.44 |
|  | N2 | 4.20±0.34 | 6.43±0.44 | 1.92±0.15 | 12.55±0.60 |
|  | N3 | 2.02±0.21 | 5.34±0.29 | 2.09±0.18 | 9.45±0.19 |
| **Female** | N1 | 6.20±0.50 | 6.98±0.28 | 5.54±0.10 | 18.73±0.43 |
|  | N2 | 5.86±0.41 | 5.25±0.75 | 2.95±0.47 | 14.05±1.56 |
|  | N3 | 2.81±0.10 | 3.50±0.13 | 3.04±0.09 | 9.35±0.11 |
| ***P* value** | Sex | *** | * | *** | * |
|  | N | *** | *** | *** | *** |
|  | Sex×N | ns | ns | *** | ns |

N1: control-nitrogen treatment, N2: limited N re-irrigated with control-nitrogen solution treatment and N3: limited-nitrogen treatment.

**Supplementary figures**

**Figure S1** The composition and relative abundance of bacterial (a) and fungal (b) communities in the rhizosphere soil, roots, old leaves and young leaves of *P. cathayana* males and females in different N treatments.

**Figure S2** The Shannon diversity of bacterial and fungal communities in different N treatments. From left to right: rhizosphere soil, roots, old leaves and young leaves. Two-way ANOVA analysis was performed. Sex: sex effect, N: N treatment effect, sex×N: sex and N interactions effect. ns: not significant, * 0.01 < *P* ≤ 0.05, ** 0.001 < *P* ≤ 0.01, *** *P* ≤ 0.001.

**Figure S3** The volcano plot illustrating the enrichment and depletion patterns of bacterial and fungal microbiomes in each niche (male vs female). Each point represents a single OTU. Each red point represents an individual enriched OTU, and a blue point represents an individual depleted OTU. The position along the y-axis represents the fold-change in abundance compared with females, and the x-axis shows the average OTU abundance (as counts per million).

**Figure S4** Different niche effects on the networks of bacterial (a) and fungal (b) communities.

**Figure S5** Phylogenetic tree, taxonomic composition, and distribution patterns of dominant fungal taxa living in roots, old leaves and young leaves. (a) Dominant fungal taxa in *P. cathayana* females and males, respectively. (b) The distribution pattern of dominant fungal taxa in *P. cathayana* females. (c) The number of dominant fungal taxa, the shared and specific bacterial OTUs in *P. cathayana* females and males. (d) The distribution pattern of dominant fungal taxa in *P. cathayana* males.

**Figure S6** Heatmaps of the expression profiles (a), differential expression analysis (DEGs) (b) and functional-enrichment analysis (KEGG) (c). MC, young male leaves in control-nitrogen treatment; MN, young male leaves in limited-nitrogen treatment; FC, young female leaves in control-nitrogen treatment; FN, young female leaves in limited-nitrogen treatment.

**Figure S1**


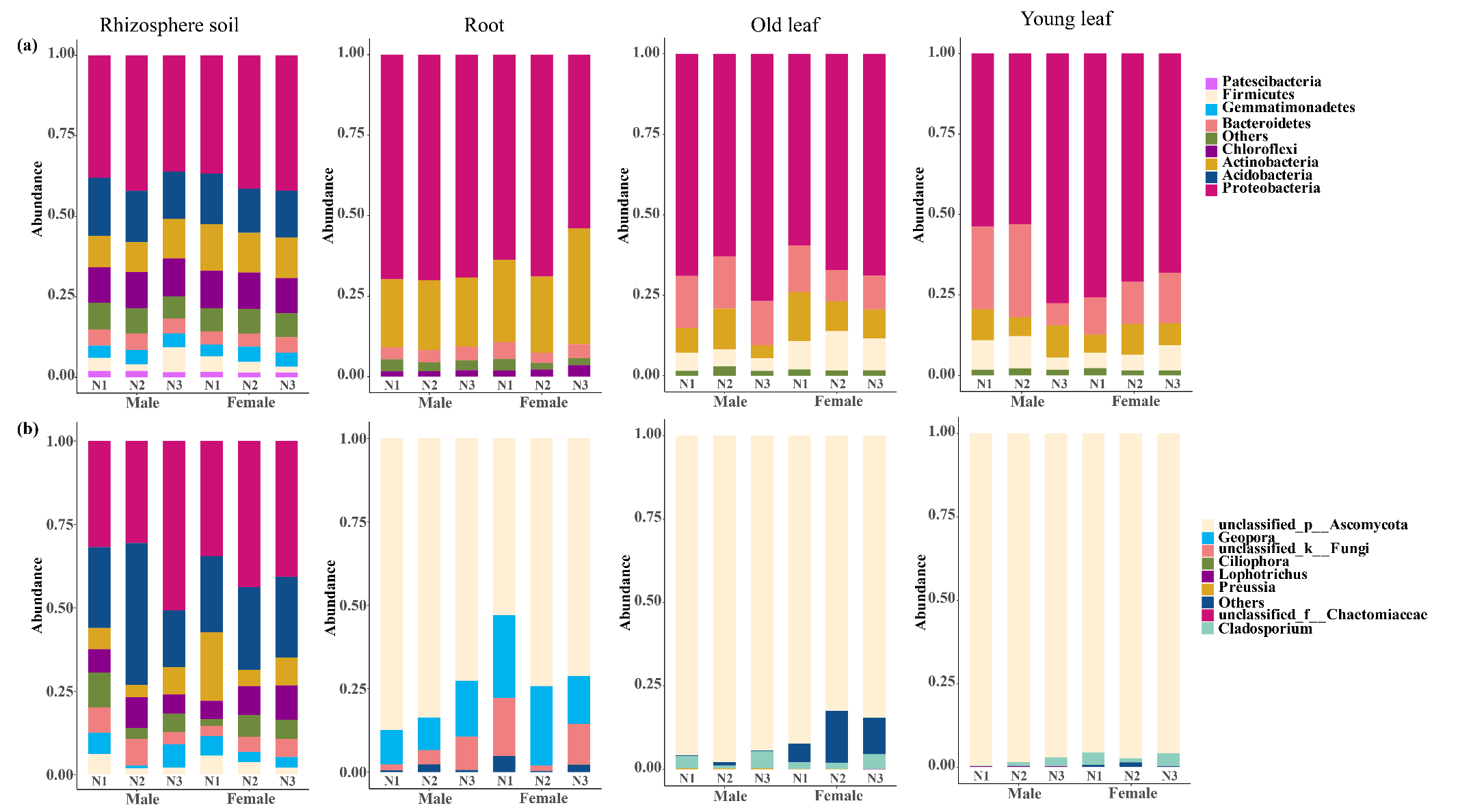


**Figure S2**


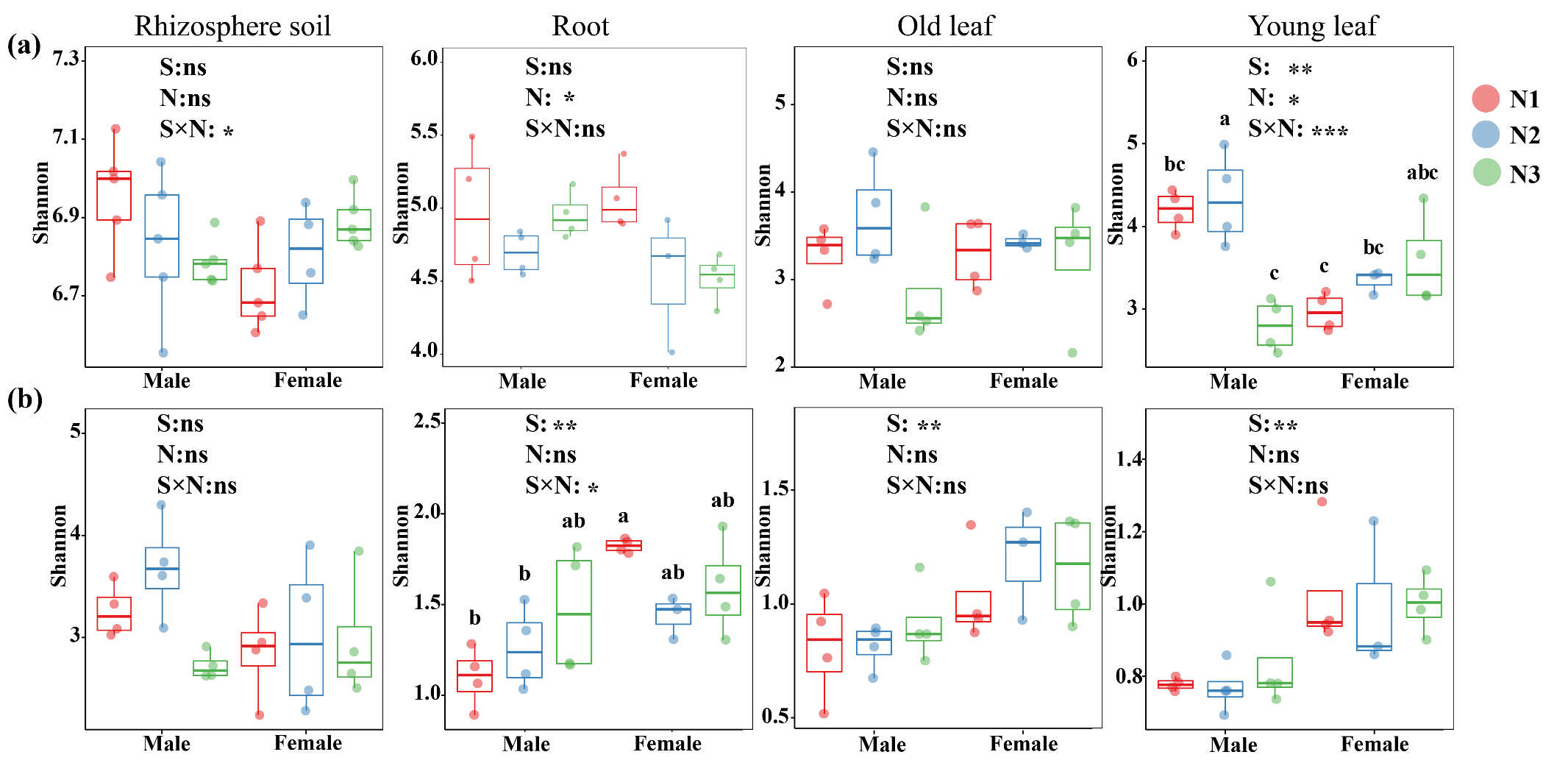


**Figure S3**


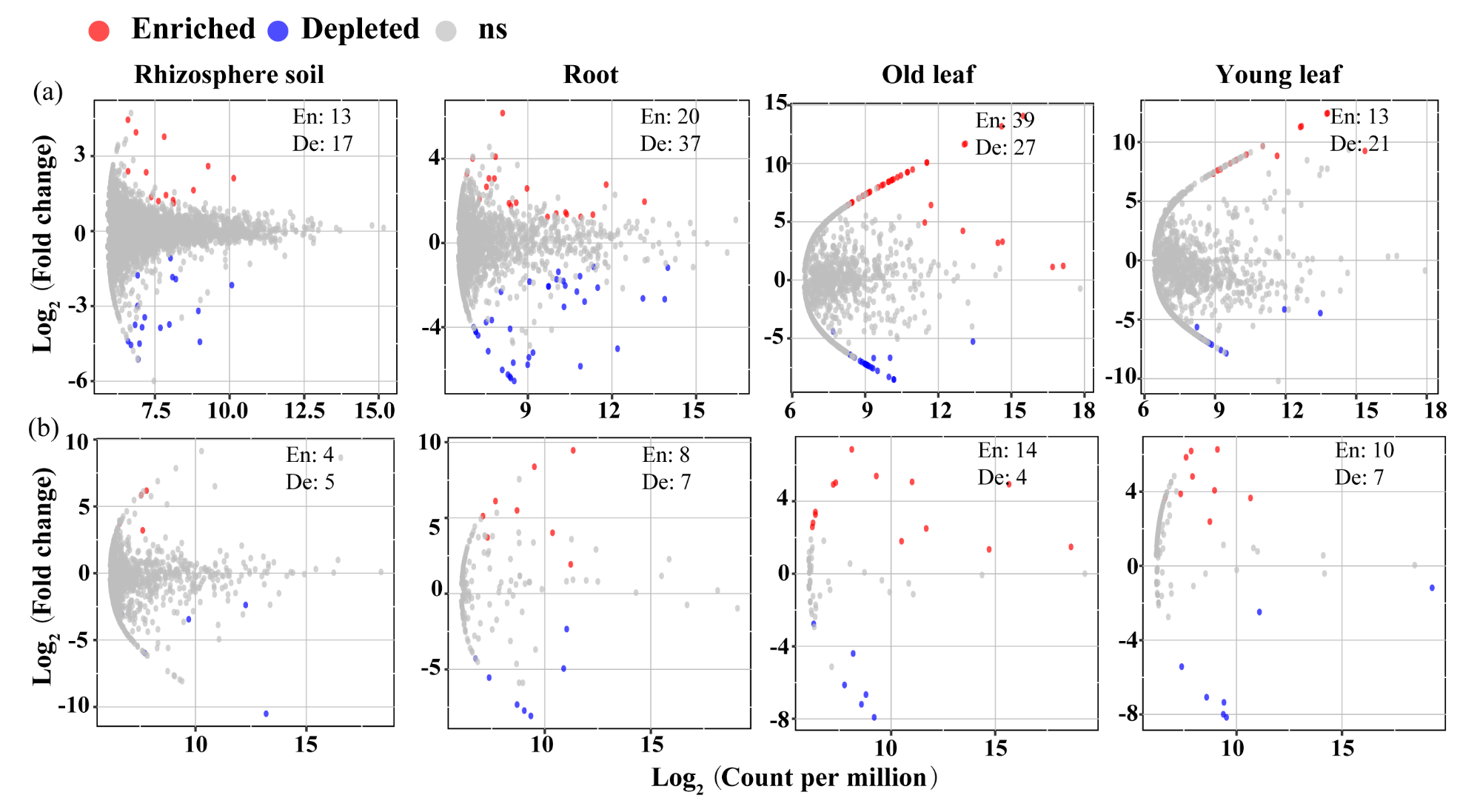


**Figure S4**


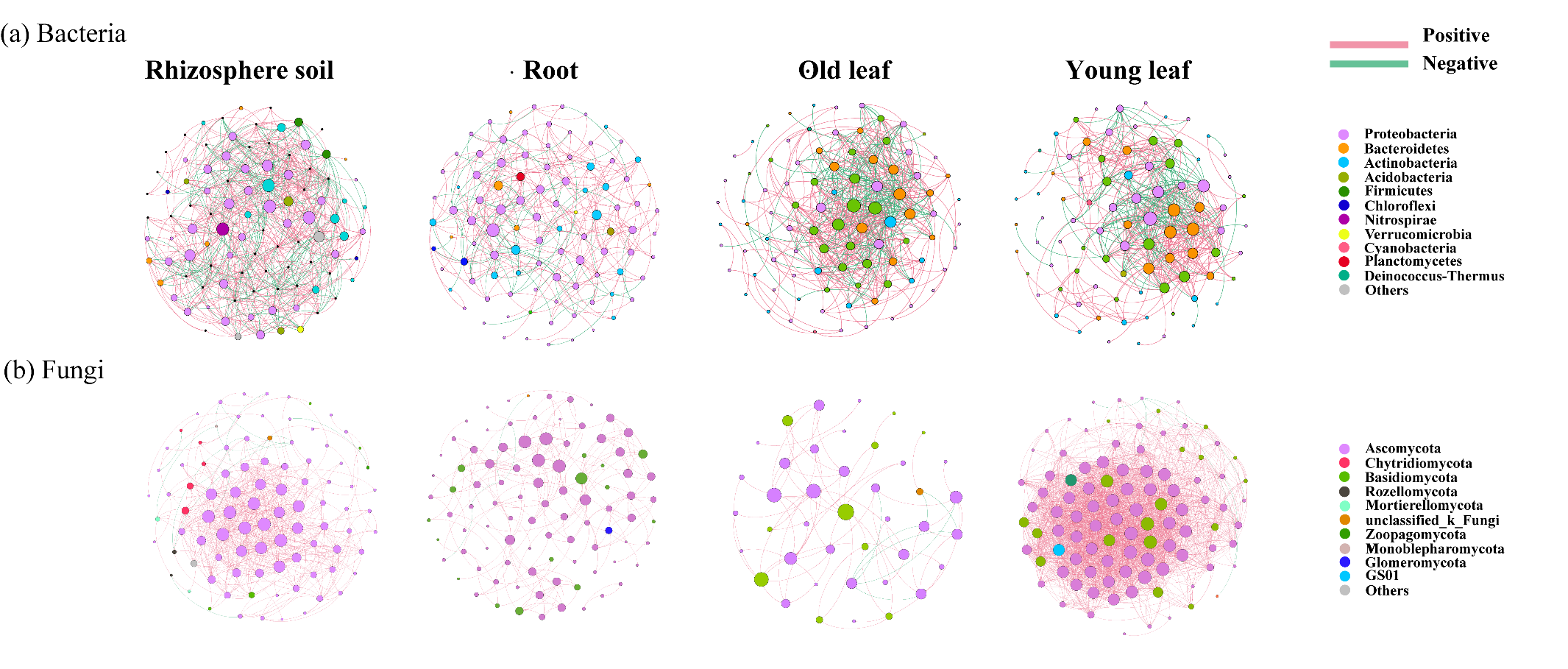


**Figure S5**


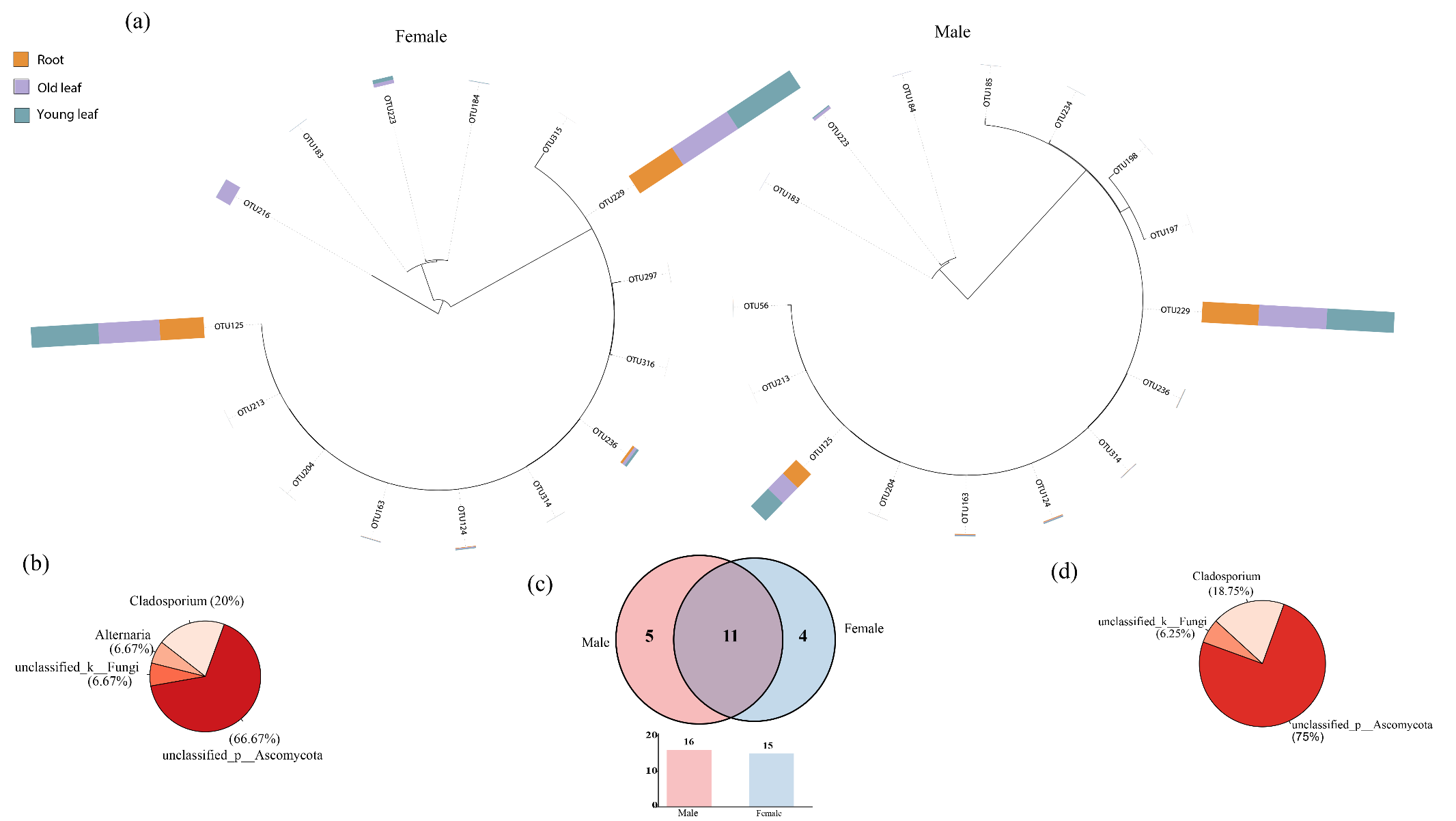


**Figure S6**


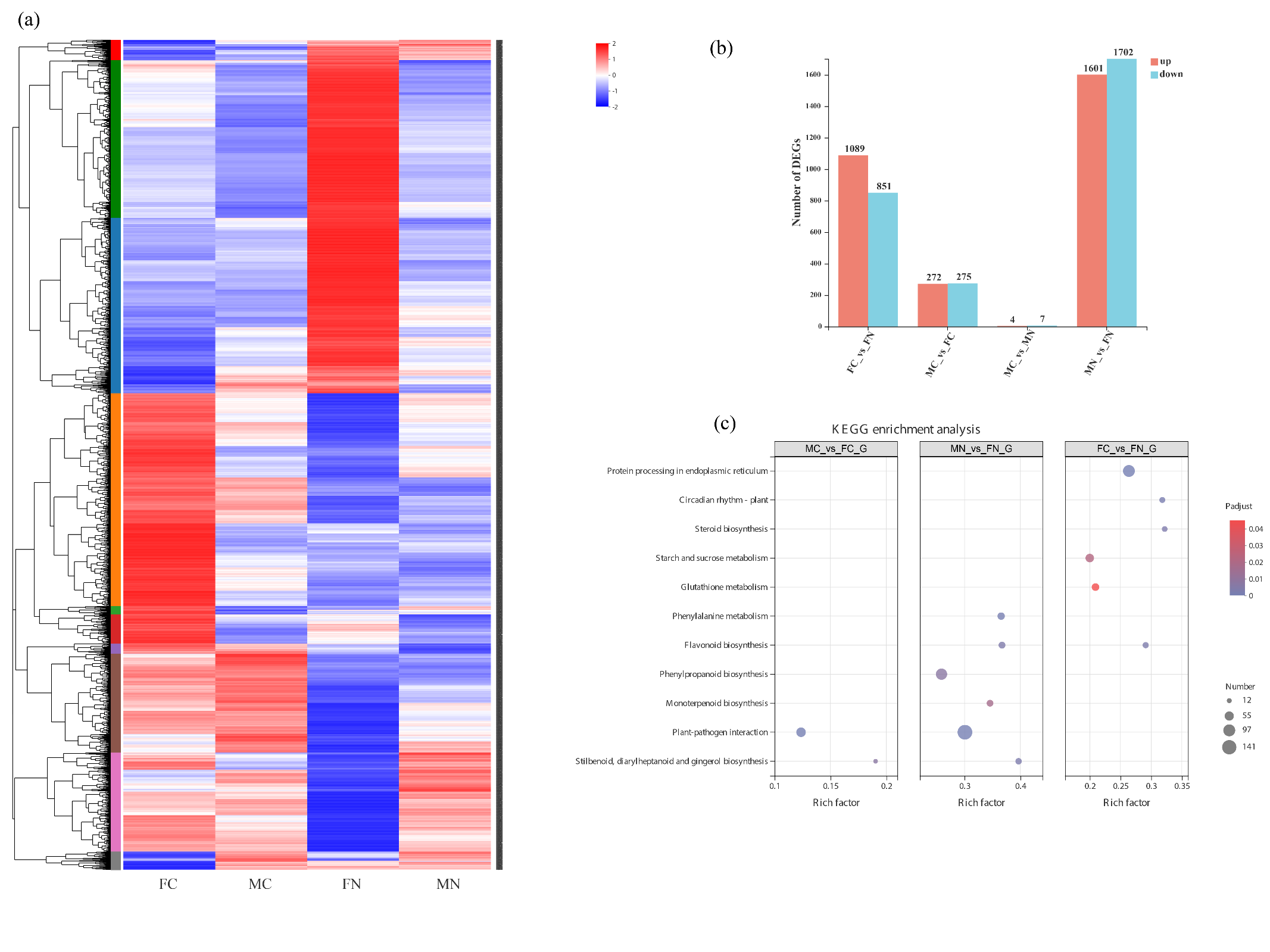

Supplement: Supplementary file 2 — Additional file 1: Supplementary methods. Table S1. Bacterial and fungal co-occurrence network characteristics in each niche. Table S2. The physiological traits and microbial functions of rhizosphere soil affected by sex and nitrogen treatment. Table S3. The growth characteristics of P. cathayana males and females under different N treatments. Figure S1. The composition and relative abundance of bacterial (a) and fungal (b) communities in the rhizosphere soil, roots, old leaves and young leaves of P. cathayana males and females in different N treatments. Figure S2. The Shannon diversity of bacterial and fungal communities in different N treatments. From left to right: rhizosphere soil, roots, old leaves and young leaves. Figure S3. The volcano plot illustrating the enrichment and depletion patterns of bacterial and fungal microbiomes in each niche (male vs female). Figure S4. Different niche effects on the networks of bacterial (a) and fungal (b) communities. Figure S5. Phylogenetic tree, taxonomic composition, and distribution patterns of dominant fungal taxa living in roots, old leaves and young leaves. (a) Dominant fungal taxa in P. cathayana females and males, respectively. (b) The distribution pattern of dominant fungal taxa in P. cathayana females. (c) The number of dominant fungal taxa, the shared and specific bacterial OTUs in P. cathayana females and males. (d) The distribution pattern of dominant fungal taxa in P. cathayana males. Figure S6. Heatmaps of the expression profiles (a), differential expression analysis (DEGs) (b) and functional-enrichment analysis (KEGG) (c). MC, young male leaves in control-nitrogen treatment; MN, young male leaves in limited-nitrogen treatment; FC, young female leaves in control-nitrogen treatment; FN, young female leaves in limited-nitrogen treatment. [file 40168_2022_1387_MOESM1_ESM.docx]
